# Effects of mediterranean diet on lung function in smokers: a randomised, parallel and controlled protocol

**DOI:** 10.1186/s12889-015-1450-x

**Published:** 2015-01-31

**Authors:** Mar Sorlí-Aguilar, Francisco Martín-Luján, Antoni Santigosa-Ayala, Josep Lluís Piñol-Moreso, Gemma Flores-Mateo, Josep Basora-Gallisà, Victoria Arija-Val, Rosa Solà-Alberich

**Affiliations:** Unitat de Suport a la Recerca Tarragona - Reus, Institut Universitari d’Investigació en Atenció Primària Jordi Gol (IDIAP Jordi Gol), Tarragona, Spain; Centre d’Atenció Primària Jaume I, Direcció d’Atenció Primària Tarragona, Institut Català de la Salut, Tarragona, Spain; Centre d’Atenció Primària Sant Salvador, Direcció d’Atenció Primària Tarragona, Institut Català de la Salut, Tarragona, Spain; Direcció d’Atenció Primària Tarragona, Institut Català de la Salut, Tarragona, Spain; Facultat de Medicina i Ciències de la Salut, Universitat Rovira i Virgili, Reus, Spain

**Keywords:** Mediterranean diet, Smoking, Lung function, Randomized controlled trial, Primary health care

## Abstract

**Background:**

There is evidence of an association between pulmonary function and various nutrients, although no association has been observed in our setting between the Mediterranean Diet (MD) eating pattern and improved lung function. The objective of this study is to evaluate the effect of an intervention designed to increase MD adherence on lung function in smokers with no previous respiratory disease.

**Methods/design:**

Randomized, controlled, parallel clinical trial. Setting: primary health care centers in Catalonia (Spain). Participants: Current smokers (cumulative > 10 pack-years) aged 35–70 years, with Internet access, who provide signed informed consent to participate. Intervention: A nutritionist will conduct a 2-year multicomponent intervention to increase MD adherence, based on: 1) a personalized dietary-nutritional education intervention, 2) a Web 2.0 approach, the DIET Blog of nutritional information, and 3) group sessions to increase motivation to increase MD adherence and motivation to make changes in eating habits. Annually, an office visit and one group session will reinforce the nutritional intervention. The control group will follow their usual diet, with general nutritional counselling. In both groups, a 14-item questionnaire will evaluate individual MD dietary patterns and forced spirometry will assess lung function. Analysis: Intention to treat. The unit of analysis will be the individual smoker. Primary outcome is lung function indicated by spirometry, FVC, FEV1 and FEV1/FVC %. Lung function parameters in both groups will be compared by adherence to the MD pattern.

**Discussion:**

The DIET study could contribute data on a protective action of the MD pattern on lung function in smokers. If so, this population may benefit from a nutritional intervention, along with the fundamental recommendation to stop smoking.

**Trial registration:**

ClinicalTrials.gov: NCT02151669. Registered 26 May 2014.

## Background

Diseases of the respiratory system are a public health problem of the highest order [1]. Although smoking is the major risk factor for respiratory disease, other factors such as environmental agents, respiratory infections, genetic disorders and eating habits may also affect lung function [2].

In our setting, smoking is the main cause in the etiopathogenesis of respiratory disease [3]. Inhalation of the particles found in tobacco smoke accelerates the age-related physiological decline of pulmonary capacity [4]. It has been estimated that each cigarette smoked exposes the smoker to 10^15^ free-radical particles, a major source of oxidative stress and inflammation [5]. To counteract this effect, the first line of defence is that provided by several enzymatic antioxidants: superoxide dismutase, catalase and peroxidase glutathione. It has also been suggested that natural antioxidants such as vitamin C, vitamin E and ß-carotene could have a protective effect on lung function [6]. In fact, this is not a new idea. There is evidence of protective action by certain foods and nutrients on lung function parameters such as forced vital capacity (FVC) and forced expiratory volume in 1 second (FEV_1_). Along these lines, daily consumption of fruits and vegetables has been inversely associated, independently of adjustments for total calorie intake and body mass, with chronic obstructive pulmonary disease (COPD) [7,8]. Fibre intake has also shown an inverse relationship with the development of COPD in long-term observational studies [9,10]. Omega-3 fatty acids found in certain fish and seafood may also help to prevent lung disease. A cross-sectional study of the ARIC cohort has demonstrated the protector effect of these dietary elements in smokers and exsmokers with COPD [11]. The relationships between lung function and saturated fats, or polyunsaturated fats, suggest that some of the inflammatory mechanisms of atherosclerosis could be involved in the physiopathology of COPD and some researchers have even suggested the use of cholesterol-lowering medications such as statins in COPD for their anti-inflammatory effect [12].

At present, however, the analysis of dietary patterns is considered the best approach in the study of the health effects of food intake, rather than a focus on specific foods or nutrients, because the diet as a whole incorporates all of the synergistic effects of all foods consumed [13]. In a recent U.S. study with a 3 –year follow-up, the eating habits of 15,567 participants were assessed using a healthy eating index (HEI-2005). The authors describe a significant positive association between lung function and consumption of omega-3 polyunsaturated fatty acids and dietary fibre and a negative association with total intake of calories and saturated fatty acids [14]. The traditional dietary pattern in Mediterranean countries, the Mediterranean Diet (MD), encompasses a combination of characteristic foods such as fruits, vegetables and fresh produce, fish and seafood, nuts, legumes/pulses and olive oil. These foods are rich in antioxidants such as vitamin C, vitamin E, ß-carotene, phytoestrogens, folates or selenium and mono- and polyunsaturated fatty acids, and have a low percentage of saturated fats and cholesterol. This MD pattern has been associated with large reductions in the incidence of major cardiovascular events and in all-cause mortality [15,16].

Although it would obviously be of interest if this dietary pattern could be recommended for purposes other than the prevention of cardiovascular diseases, insufficient empirical evidence is available to establish improved lung function in smokers with better MD adherence [17].

The aim of the study is to evaluate the effects of a nutritional education intervention designed to increase adherence to the MD pattern, compared to the participant’s usual diet, on lung function in smokers with no previous respiratory disease.

## Methods

### Study design

**Figure 1:**
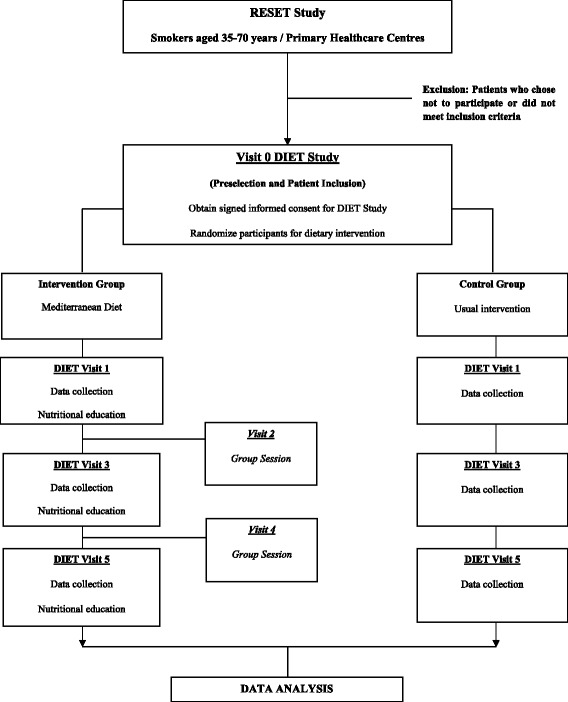
**Flowchart of the study: participant selection, randomization and follow-up.**

The DIET study (Diet, Spirometry and Tobacco) is a randomized, controlled, parallel clinical trial to be carried out in a subsample of participants in the RESET-DIET project. The main project was designed in two phases. The first phase is the RESET study (FIS-2011, PI11/01962), a randomized, controlled, multicentre clinical trial involving current smokers aged 35 to 70 years with no history of respiratory disease. This study aims to evaluate the effectiveness of a motivational intervention based on spirometry information to promote smoking cessation [18]. Participants who are still smoking at the end of the RESET follow-up will be candidates to participate in the second phase: the DIET study [19] (Figure 1: Flowchart of the study).

### Participants

#### Selection of participating primary care centers

Primary health care centers of Reus city (Tarragona) and surrounding areas.

#### Randomization

Individuals who provide signed consent to participate will be randomized (1:1) to two study groups, intervention and control. The groups will be assigned consecutively in a centralized process carried out by the Research Support Unit of the Institut d’ Investigació en Atenció Primària (IDIAP) Jordi Gol, following a simple random number list computer-generated for this purpose using EPIDAT 3.0.

##### Patient inclusion criteria

- Men and women aged 35 to 70 years
- Current smokers at the time of inclusion (defined as a cumulative smoking habit >10 pack-years, calculated as years of smoking multiplied by the average daily consumption of 20-cigarette packs)
- Internet access and ability to use new technologies, or having someone available to provide this assistance
- Signed informed consent to participate

##### Patient exclusion criteria

- Any evidence in the medical records of a history or diagnosis of any type of respiratory disease
- Any chronic or terminal condition that could affect baseline parameters or make it difficult to complete the tests required during the study period
- Inability to participate in the follow-up for any reason
- Declined to continue study participation

### Intervention

Candidates for study participation will be informed about the DIET study and offered an opportunity to participate in participant selection and randomization (visit 0). Those who accept the invitation and remain eligible after implementing the inclusion/exclusion criteria will be asked to provide signed informed consent and will then be randomly assigned to the control or intervention group. All of the interventions will be carried out by the DIET project nutritionist.

The intervention group participants will begin a Mediterranean Diet Nutritional Education Program specifically designed to increase adherence to the MD pattern. The program will have three components: 1) Individual intervention – a personalized 1-hour visit (visit 1) to explain the MD pattern and the benefits of following it. The main objective will be to modify the dietary pattern rather than to focus on changes in specific foods or micronutrients. The 14-item MD Adherence Questionnaire will be used to assess the implementation of the intervention, support provided, and fulfilment of project objectives. The questionnaire assigns one point to each item and classifies MD adherence as low, moderate or high on the basis of the final score. Educational and visual materials will also be used, such as the Food Pyramid or food groups adapted to the personal habits and preferences of each participant. 2) During this same initial visit, the participant will be given access to the DIET study’s nutritional blog, which will contain information about the MD and its benefits, cooking habits, modified recipes, and other topics of interest. The DIET Blog information will be updated periodically. 3) Finally, with the goal of increasing MD adherence and motivation to make changes in eating habits, participants will be invited to a 30-minute group session. The sessions will highlight the principal MD components and will complement the nutritional education session with information about healthy cooking techniques and about reading and understanding nutritional labels. In addition, participants will have the opportunity to ask questions about following the dietary pattern and about the advice they have received.

All participants will receive two years of follow-up. They will be invited to schedule an annual follow-up visit (visits 3 and 5, Figure 1), which will be the same as visit 1 for the group to which they were randomly assigned. Additionally, participants in the intervention group will be invited to an annual group session (visits 2 and 4, Figure 1) midway between the follow-up office visits.

### Control group

In a visit lasting about 30 minutes (visit 1), control group participants will provide the necessary study data and receive written dietary recommendations appropriate to their clinical-metabolic situation (advice to control weight, glycaemia, cholesterol, blood pressure, etc.). These patients will then be directed to their family nurse and/or doctor to complete the study’s medical testing: spirometry, 12-lead electrocardiogram and blood tests.

### Measures

All participants will be contacted by telephone to schedule the **inclusion visit (visit 1)**, which will include the standardized medical tests and completion of a data collection questionnaire containing the following information:

- Sociodemographic and family data: age, sex, ethnicity, marital status, number of children, education level, socioeconomic status, employment
- History of diseases and respiratory symptoms
- Tobacco use: current number of cigarettes/day), cumulative consumption (packs/year) and age when the participant started smoking
- Dietary habits and lifestyle
  - Alcohol use, measured in units/week (one unit = 10 gr alcohol)
  - Food Frequency Questionnaire [20]
  - Questionnaire on MD Adherence (14 points) [21]
  - Minnesota Leisure Time Physical Activity Questionnaire, validated for the population of Spain [22]
- Basic physical examination, including weight (Kg), height (cm), body mass index (BMI = Kg/m^2^), waist circumference (cm) and blood pressure (mmHg)
- Exhaled carbon monoxide measured by carboximetry
- 12-lead electrocardiogram
- Basic blood tests to measure glucose, triglycerides, cholesterol (total, high-density lipoprotein and low-density lipoprotein, creatinine and transaminases (aspartate-aminotransferase and alanine-aminotransferase).
- Forced spirometry and bronchodilation test to obtain FVC and FEV_1_ values and the FEV_1_/FVC% ratio, following current guidelines [23].

The primary outcome variable to determine whether the objective has been met will be the change in lung function indicated by spirometry, FVC, FEV_1_ and FEV_1_/FVC %. The secondary outcomes variables will be changes in MD pattern, anthropometric measurements (weight, BMI and waist circumference) and smoking habit.

### Sample size/power calculation

A sample of 40 participants in each group will be required to observe significant differences in lung function between groups (decrease in FEV_1_ > 10–12 ml/year), in a two-tailed test with a mean FEV_1_ value of 95%, significance level of 5%, 80% strength and less than 15% losses to follow-up [24].

### Statistical analysis

The study will use an intention-to-treat analysis and the main outcome variable will be measured at the beginning of the study for each participant. The effectiveness of the simple randomization will be evaluated by the comparability and homogeneity of the two groups in terms of the baseline distribution of the study variables. Losses to follow-up will be calculated for each group and evaluated to determine whether the proportion of losses is independent of assignment to the intervention or control group.

Qualitative variables will be described as frequencies or percentages and quantitative variables by means and standard deviations if they follow a normal distribution, or median and interquartile range.

Variables will be stratified by study group; basal comparison will be done by x^2^ test for qualitative variables and Student t or Mann–Whitney U for quantitative variables. Data from the initial MD Adherence Questionnaire and Food Frequency Questionnaire (V1) will be compared to the final questionnaire data (V5). In addition, changes from baseline in lung function after the corresponding nutritional intervention, will be compared for both groups. If important clinical differences are observed, multivariate analysis will be used to determine which factors are independently associated with the outcomes, adjusting for covariates such as age, sex, ethnic group, height, BMI, education level, smoking habit, physical activity or total caloric intake [6]. The results will be presented using relative measures including relative risk (RR) and relative risk reduction (RRR), and absolute risk reduction (ARR), with 95% confidence intervals. All analysis will be done with SPSS version 22.0, considering differences to be significant when *p* < 0.05.

### Ethical aspects of the study

The study will be carried out in accordance with the principles of the revised Declaration of Helsinki and the pertinent clinical guidelines for Spain (*Buena Práctica Clínica*) [25,26]. The protocol and all pertinent documents have been evaluated and approved by the committee on clinical research ethics (*Comité Ético de Investigación Clínica CEIC)* of the Institut d’Investigació en Atenció Primària (IDIAP) Jordi Gol. The project also complies with Spain’s relevant legislation on data privacy and biomedical research (*Ley Orgánica de Protección de Datos de Carácter Personal, 15/1999 del 13 de diciembre; Ley 14/2007 de Investigación biomédica, del 3 de julio*).

## Discussion

The DIET project results have the potential to contribute new data on the protective action of MD adherence for lung function in smokers with no history of respiratory disease. If a beneficial effect is confirmed, this population could benefit from specifically targeted nutritional intervention, together with the fundamental recommendation, which is to stop smoking.

As has been suggested in the literature, the combined action of different elements of the MD could provide an added functional benefit to individuals with increased risk of respiratory impairment, COPD, and death [6]. Therefore, the objective of the present study is to evaluate the effectiveness of an intervention designed to increase adherence to the MD pattern in smokers with no history of respiratory disease as a preventive strategy to reduce the harm produced by exposure to various oxidant elements of tobacco smoke.

Although the literature contains evidence of a positive impact of the intake of specific nutrients on various parameters of lung function [6-11], studies of particular foods or nutrients do not represent overall food consumption and therefore the epidemiological information provided may not reflect reality. For that reason, the hypothesis of dietary patterns is more prevalent because both foods and nutrients have synergistic and antagonistic effects when consumed in different combinations [27]. In this sense, the cardioprotector effect of the traditional MD pattern is being studied because of the multiple benefits observed in total as well as cardiovascular mortality [28], but a beneficial effect has not yet been described in respiratory disease.

The present study has several limitations. The most important is related to the study population, because the inclusion criteria may limit successful recruitment. Only RESET study participants who complete the follow-up and have the required spirometric and nutritional data will be considered for the DIET study. The intervention design is another potential limitation because it uses a new strategy in the field of respiratory disease prevention and smoking cessation. Although it may appear to be less intensive than what is described in the cardiovascular prevention literature [29], this is a pragmatic study that takes into account the actual working conditions in daily clinical practice in primary health care.

The theoretical framework is the provision of personalized and motivational information about the benefits of adopting a dietary pattern based on the MD. Recent studies have shown that one of the major aspects of this model of healthy eating is that is based on diet quality more than on the relative quantities of specific nutrients consumed [30]. Therefore, the program focuses on two major areas of emphasis: 1) reinforcement of the consumption of foods belonging to the traditional MD pattern (also known as a *prudent* diet) and considered protective because of their nutritional composition, including virgin olive oil, nuts, legumes/pulses, salads, fruits and vegetables, whole grains, fibre-rich foods, and fish, and 2) decrease consumption of foods whose nutritional composition has negative effects (also known as a *Western* diet), such as fast food, commercially prepared foods, sausages or processed meats and patés, refined sugars and flours, sugary drinks, French fries, transfats (e.g., commercial pastries), sweets and desserts [31]. Each participant in the intervention group will receive guidance based on his or her scores from the 14-point MD Adherence Questionnaire and Food Frequency Questionnaire. The annual administration of these instruments, validated for the population of Spain and used in other prospective studies [16], will allow us to evaluate the level of MD adherence as well as changes during follow-up, ensuring a high-quality measurement of a participant’s actual diet. Nevertheless, a potential study limitation in the subjective nature of the information obtained. A possible line of future research will be the measurement of biomarkers of the MD pattern such as the level of oleic and alpha- linoleic acids, reliable indicators of an individual’s intake of monounsaturated and polyunsaturated fatty acids, respectively, and urinary concentrations of tyrosol and hydroxityrosol (olive oil) and resveratrol and ethanol (wine and alcoholic drinks in general).

With the goal of minimizing the possible losses to follow-up, a common concern in long-term projects, and increase the motivation of study participants, a multicomponent intervention has been designed. The use of new technologies has proven useful in health care settings, reducing costs and improving treatment adherence [32]. The creation of the DIET Blog as a nutritional tool 2.0 to reinforce the nutritional education program is intended to increase MD adherence during follow-up. In addition to making recipes and educational materials available periodically, the blog will provide an ongoing “reminder” of the benefits of a balanced diet and facilitate a more agile and flexible interaction between participants in the intervention group and the dietician, beyond their consults in the primary care centre.

With respect to group sessions, the third component of the intervention design, these will be more recreational. In addition to reinforcement of the main items that are characteristic of the MD pattern and the opportunity to clear up any questions or misunderstandings, the sessions will encourage participants to exchange experiences, raise awareness and increase motivation. This will affect the change in attitudes that is absolutely necessary to initiate changes toward eating habits that are healthy and balanced, along with other lifestyle habits such as physical exercise, smoking cessation, etc.

Knowledge and skills are essential to properly evaluate MD adherence in terms of specific foods, as well as to increase the validity and reliability of the information collected using the study questionnaires. Therefore, the expertise of an experienced, highly qualified nutritionist is a fundamental requirement for the success of the nutritional intervention and participant follow-up.

The effect on lung function will be evaluated by comparing the results of three consecutive forced spirometry measurements, taken annually during the three-year project period. Although in theory this time period is sufficient to allow observations about the longitudinal effects of the diet on spirometry values, it could be too short to show statistically and clinically significant differences between the two groups. In any case, these results should provide additional evidence of the effect of a Mediterranean-type dietary pattern on lung function in a population of smokers with no previous respiratory disease.
